# Trends and sex disparities in school bullying victimization among U.S. youth, 2011–2019

**DOI:** 10.1186/s12889-020-09677-3

**Published:** 2020-10-21

**Authors:** Ruili Li, Qiguo Lian, Qiru Su, Luhai Li, Meixian Xie, Jun Hu

**Affiliations:** 1 Children Health and Development Department, Capital Institute of Paediatrics, Beijing, China; 2 NHC Key Lab. of Reproduction Regulation (Shanghai Institute of Planned Parenthood Research), Fudan University, Shanghai, China; 3 Shenzhen Children’s Hospital, Shenzhen, Guangdong China; 4 Wanrong Maternal and Child Care Service Centre, Wanrong, Shanxi China; 5 Huajing Community Health Service Center, Shanghai, China

**Keywords:** School bullying, Sex disparities, Trends

## Abstract

**Background:**

The prevalence of being bullied traditionally among U.S. high school students is expected to reduce to 17.9%, according to Healthy People 2020 Initiatives. We examined trends in traditional victimization and cybervictimization with the latest large-scale time-series data in the United States.

**Methods:**

We analyzed the data from the 2011–2019 national Youth Risk Behavior Survey (YRBS) to access the trends in traditional victimization and cybervictimization among U.S. high school students. We identified the temporal trends using multivariate logistic regression analyses, accounting for survey design features of YRBS. Participants included 72,605 high school students.

**Results:**

The overall prevalence of victimization was 19.74% for traditional bullying and 15.38% for cyberbullying, suggesting that cyberbullying is not a low frequent phenomenon. The prevalence of victimization ranged from 20.19 to 19.04% for traditional victimization and 16.23 to 14.77% for cybervictimization, and the declined trends for the two kinds of bullying victimization were both statistically non-significant. The degree of overlap between the two kinds of bullying victimization was about 60%. Besides, female students reported more traditional victimization and cybervictimization than male peers within each survey cycle.

**Conclusions:**

No declined trends in traditional victimization and cybervictimization were observed during 2011–2019. Female students are more likely to experience school bullying. To achieve the Healthy People 2020 goal on bullying, more work is needed to explore the underlying reasons behind these unchanging trends.

## Background

School bullying was considered a serious and often overlooked issue for school-aged children [1]. There are four main forms of bullying: physical, verbal, social, and cyber. Globally, more than 10% of the students reported being bullied in school at least 2–3 times a month according to the 2010 Health Behaviour in School-aged Children (HBSC) study [2]. Bullying victimization is positively associated with mental health problems including depression [3], self-harm [4] and suicidal ideation [5], and externalizing problems [6].

Prof. Dan Olweus of Norway, one of the world’s pioneering researchers on bullying, provided strong evidence that compared with traditional verbal bullying(17.3%), cyberbullying(4.5%) was still a relatively low-prevalence phenomenon, which didn’t increase over time and the degree of overlap with traditional bullying was very high (almost 90%) using large-scale time-series data sets in United States(2007–2010) [7, 8]. The heterogeneity of empirical prevalence estimates of cyberbullying, according to Olweus, can be can be explained by three primary reasons: different reference periods, different cutoff points, and different measurement contexts [9].

Cyberbullying has evolved with the rapid development of technology and social media in the past decade. The trend of cyberbullying victimization during recent years has been poorly studied although traditional bullying has begun to decline among adolescent populations [10]. The 2002–2014 HBSC data in 37 countries suggested linear decreases or no linear trends in traditional victimization in most countries, and a moderate degree of overlap (45.8%) of traditional victimization and cybervictimization with considerate country variations [11]. However, the previous research failed to examine the trend of cyberbullying victimization because cyberbullying items were introduced to HBSC since 2014.

Given the highly inconsistent findings on trends and degree of overlap in bullying [9, 11, 12] and the rapid evolution of social media, it’s necessary to address this gap using the latest time-series data measuring both traditional victimization and cybervictimization in a national representative sample. Leveraging the data from the 2011–2019 U.S. Youth Risk Behavior Survey (YRBS), our first objective was to estimate the prevalence and the degree of overlap, and examine the trends in traditional victimization and cybervictimization among U.S. high school students. Our second objective was to evaluate the Healthy People 2020 Objective IVP-35, the prevalence of traditional bullying victimization among U.S. high school students was expected to reduce to 17.9% in YRBS [13].

## Methods

### Data and participants

The YRBS is an ongoing, biennial, cross-sectional, school-based survey of a representative sample of high school students from across the U.S. that monitors the prevalence of health-related behaviors [14, 15]. A three-stage cluster sample design is used in YRBS to recruit nationally representative samples of students attending public and private schools in grades 9–12 [14, 15]. The anonymous survey uses a computer-scannable questionnaire and takes about 45 min [14, 15].

This study drew data (*n* = 72,605) from five cycles of YRBS (2011–2019), considering that YRBS measured both traditional bullying and cyberbullying since 2011. The overall response rates remained at > 60% during 2011–2019 [14, 15]. The data used in this secondary analysis are de-identified and publicly available (https://www.cdc.gov/healthyyouth/data/yrbs), hence no protocol approval from an institutional review board was needed.

### Measures

The YRBS measured traditional victimization and cybervictimization using the same reference period (in the past 12 months). During 2011–2019 YRBS, traditional victimization was assessed by the question “During the past 12 months, have you ever been bullied on school property?” (No = 0, Yes = 1). During 2011–2015 surveys, cybervictimization was assessed by the question “During the past 12 months, have you ever been electronically bullied? (Include being bullied through e-mail, chat rooms, instant messaging, websites, or texting.)” (No = 0, Yes = 1). In the 2017–2019 YRBS, the definition of cybervictimization was revised to “Count being bullied through texting, Instagram, Facebook, or other social media”. We hence derived a variable to represent three forms of bullying victimization: traditional victimization only, cybervictimization only, and polyvictimization. The degree of overlap between traditional victimization and cybervictimization was measured as the ratio of polyvictimization to cybervictimization, indicating the proportion of participants who experienced cybervictimization also experienced traditional victimization [8, 11].

The YRBS included two questions about race and Hispanic heritage since 2007: “Are you Hispanic or Latino?” (Yes = 1, No = 2); What is your race? (Select one or more responses.) (American Indian or Alaska Native = 1, Asian = 2, Black or African American = 3, Native Hawaiian or Other Pacific Islander = 4, White = 5). Based on the two aforementioned questions, we computed a 4-level race/ethnicity variable (White = 1, Black or African American = 2, Hispanic/Latino = 3, All Other Races = 4).

We generated grade variable from the question: “In what grade are you?”(9th grade = 1, 10th grade = 2, 11th grade = 3, 12th grade = 4), and sex variable from the question: “What is your sex?” (Female = 1, Male = 2).

### Data analysis

Data were weighted to account for the complex survey design and adjusted for the survey nonresponse. Unweighted sample sizes were presented along with weighted prevalence estimates and corresponding 95% confidence intervals (CIs). Chi-squared tests of independence were used to examine statistical differences between female and male students across survey years. The trends in traditional victimization and cybervictimization during 2011–2019 by different sex groups were examined using logistic regression models, adjusting for sex, race/ethnicity, and grade [16]. Survey year was used as a continuous variable to assess the linear trend, and quadratic terms of survey year were included to examine the quadratic trend. Only linear time variable was included in the logistic regression models to examine the linear trends, both linear and quadratic time variables were included when examining the quadratic trends [17]. Odds ratios(ORs) and 95% CIs were reported. Given than the proportion of missing data in this study was very small (traditional victimization: 1.80% on average, cybervictimization: 2.88% on average), we used pairwise deletion method to deal with missing data.

All analyses were performed using SVY procedures in Stata/SE 15.1 (StataCorp LLC). Statistical significance was considered if a 2-tailed *p*-value was less than 0.05.

## Results

Of the 72,605 participants from five survey cycles of YRBS, there were 36,497(49.41%) female students and 361,08(50.59%) male students (Table 1). The sample was racially/ethnically diverse and largely composed of White, non-Hispanic students (54.40%).

**Table 1:** Sample Characteristics of high school students: YRBS 2011–2019

Characteristic	No. (Weighted %)		
	Overall	Male	Female
All respondents	72,605 (100)	36,108 (100)	36,497 (100)
Grade			
9th	18,868 (27.26)	9284 (27.63)	9584 (26.87)
10th	18,156 (25.68)	9001 (25.67)	9155 (25.68)
11th	18,127 (23.98)	9145 (23.89)	8982 (24.07)
12th	17,042 (23.09)	8426 (22.80)	8616 (23.38)
Race/ethnicity			
White, non-Hispanic	31,329 (54.40)	15,601 (54.31)	15,728 (54.48)
Black, non-Hispanic	12,236 (13.55)	6020 (13.67)	6216 (13.42)
Hispanic	19,781 (22.42)	9777 (22.38)	10,004 (22.46)
Other, non-Hispanic	7787 (9.64)	3866 (9.64)	3921 (9.63)
Survey cycle (year)			
2011	15,364 (21.16)	7656 (21.56)	7708 (20.74)
2013	13,571 (18.68)	6950 (18.47)	6621 (18.89)
2015	15,506 (21.34)	7749 (21.64)	7757 (21.04)
2017	14,638 (20.17)	7112 (19.65)	7526 (20.69)
2019	13,526 (18.65)	6641 (18.67)	6885 (18.64)

During 2011–2019, 19.70% of the U.S. high school students reported traditional victimization, and 15.44% reported cybervictimization, female students reported higher prevalences of both traditional victimization and cybervictimization than male students (Table 2). Among high school students, 10.11% were bullied traditionally only, 5.71% were cyberbullied only, and 9.73% were bullied in both ways. Female students reported higher prevalences of both cyber only and poly forms than male students. We further stratified the analyses by survey year and produced consistent results.

**Table 2:** Weighted percentage of school bullying victimization by survey cycle and sex: YRBS 2011–2019

Survey cycle and sex	School bullying forms			School bullying victimization		Overlap
	Traditional only Weighted%(95%CI)	Cyber only Weighted%(95%CI)	Both(poly-victimized) Weighted%(95%CI)	Traditional Weighted%(95%CI)	Cyber Weighted%(95%CI)	Both/Cyber %
2011						
Overall	11.39 (10.13–12.78)	6.81 (6.25–7.41)	9.32 (8.43–10.30)	20.06 (18.61–21.59)	16.23 (15.21–17.31)	57.42
Male^***^	12.84 (11.32–14.53)	4.68 (4.03–5.44)	6.01 (4.89–7.35)	18.24 (16.52–20.10)	10.75 (9.59–12.04)	55.90
Female	9.82 (8.59–1122)	9.10 (8.27–10.01)	12.87 (11.59–14.27)	21.99 (20.42–23.64)	22.11 (20.60–23.71)	58.20
2013						
Overall	10.47 (9.79–11.19)	5.58 (5.08–6.13)	9.20 (8.41–10.05)	19.65 (18.46–20.89)	14.77 (13.68–15.92)	62.28
Male^***^	10.29 (9.26–11.41)	3.26 (2.70–3.93)	5.29 (4.69–5.95)	15.55 (14.25–16.96)	8.54 (7.70–9.47)	61.94
Female	10.63 (9.89–11.42)	7.91 (7.00–8.92)	13.11 (11.72–14.64)	23.72 (22.14–25.38)	21.01 (19.15–23.00)	62.39
2015						
Overall	10.26 (9.14–11.51)	5.61 (4.98–6.31)	9.96 (9.12–10.86)	20.19 (18.69–21.78)	15.55 (14.37–1680)	64.05
Male^***^	10.00 (8.80–11.34)	3.84 (3.21–4.59)	5.84 (4.84–7.03)	15.81 (14.42–17.31)	9.66 (8.45–1103)	60.45
Female	10.50 (9.10–12.09)	7.44 (6.52–8.48)	14.30 (12.87–15.86)	24.79 (22.75–26.96)	21.74 (19.86–23.75)	65.77
2017						
Overall	9.14 (8.44–9.90)	5.04 (4.57–5.56)	9.91 (8.99–10.91)	19.04 (17.71–20.44)	14.94 (13.77–16.18)	66.33
Male^***^	9.09 (8.13–10.15)	3.41 (2.92–3.97)	6.51 (2.85–7.25)	15.58 (14.46–16.78)	9.91 (9.19–10.68)	65.69
Female	9.26 (8.45–10.14)	6.66 (5.78–7.67)	13.09 (11.44–14.95)	22.34 (20.21–24.62)	19.74 (17.48–22.21)	66.31
2019						
Overall	9.29 (8.33–10.35)	5.50 (4.85–6.24)	10.23 (9.24–11.32)	19.52 (17.89–21.26)	15.71 (14.40–17.12)	65.12
Male^***^	8.57 (7.47–9.80)	4.14 (3.47–4.94)	6.80 (5.83–7.91)	15.36 (13.77–17.09)	10.92 (9.56–12.44)	62.27
Female	9.98 (8.80–11.29)	6.77 (5.84–7.84)	13.63 (12.23–15.17)	23.60 (21.44–25.90)	20.39 (18.63–22.27)	66.85
2011–2019						
Overall	10.11 (9.66–10.59)	5.71 (5.44–5.98)	9.73 (9.35–10.12)	19.70 (19.10–20.31)	15.44 (14.96–15.94)	63.02
Male^***^	10.18 (9.62–10.77)	3.87 (3.59–4.18)	6.08 (5.66–6.54)	16.14 (15.49–16.82)	9.96 (9.44–10.51)	61.04
Female	10.03 (9.51–10.58)	7.57 (7.15–8.01)	13.41 (12.8–14.04)	23.29 (22.47–24.13)	21.00 (20.22–21.79)	63.86

Abbreviation: *CI* confidential interval

^***^Differences in bullying among male and female students are all statistically significant (*P* < 0.001)

**Fig. 1:**
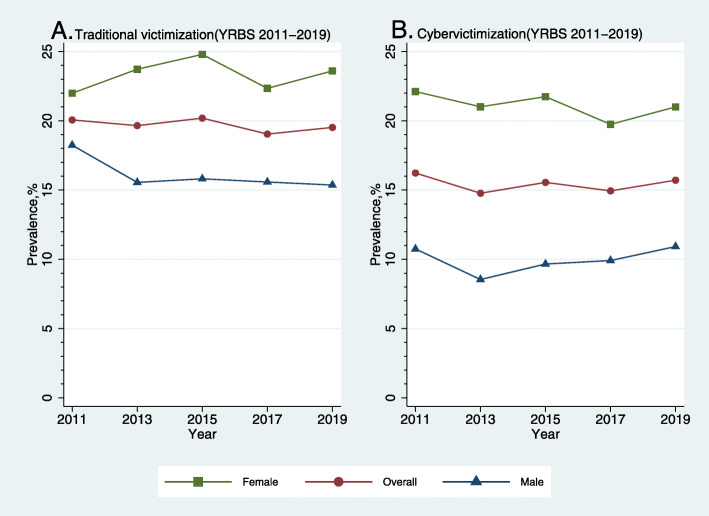
School bullying victimization trends by sex groups: YRBS 2011–2019

Linear and quadratic trend analysis showed that there were no significant changes in the prevalence of U.S. high students who reported traditional victimization or cybervictimization during 2011–2019 (Fig. 1 and Table 3). The overall prevalences were 20.06% (2011), 19.65% (2013), 20.19% (2015), 19.04% (2017), and 19.52% (2019) for traditional victimization, and 16.23% (2011), 14.77% (2013), 15.55% (2015), 14.94% (2017), and 15.71% (2019) for cybervictimization (Table 2). The sex-stratified analysis resulted in similar findings. It should be noted that although the *p* value of linear trend analysis in male students (*p* = 0.031) was less than 0.05, the 95% CI did include 1.00, hence we considered it statistically non-significant.

**Table 3:** Trends for school bullying victimization by sex groups: YRBS 2011–2019

Sex	Trends	Traditional bullying victimization		Cyberbullying victimization	
		OR (95%CI)	*P* value	OR (95%CI)	*P* value
Overall	Linear	0.99 (0.96–1.02)	0.468	0.99 (0.96–1.02)	0.471
	Quadratic	1.00 (0.97–1.02)	0.838	1.01 (0.99–1.04)	0.220
Male	Linear	0.96 (0.93–1.00)	0.031	1.02 (0.97–1.06)	0.456
	Quadratic	1.02 (0.99–1.05)	0.234	1.04 (1.00–1.08)	0.076
Female	Linear	1.01 (0.98–1.05)	0.455	0.98 (0.95–1.01)	0.113
	Quadratic	0.98 (0.95–1.01)	0.173	1.00 (0.98–1.03)	0.793

Abbreviations: *OR* Odds ratio, *CI* confidential interval

All analyses adjusted for sex, grade and race

The degree of overlap between traditional victimization and cybervictimization in our study was moderate. Overall, of students who reported cybervictimization, 63.02% also reported traditional victimization (Table 2). The overall degree of overlap ranged from 57.42% (2011) to 66.33% (2017), this degree ranged from 55.90% (2011) to 65.69% (2017) for male students, and from 58.20% (2011) to 66.85% (2019) for female students. We also observed a lower degree of overlap among male students in each survey year and the pooled sample.

## Discussion

The main findings of this study are we did not observe a downward trend in both forms of bullying victimization, and more female students reported being bullied compared with male students, both traditionally and electronically. These findings echoed the previous findings [18, 19], and highlighted the gap between reality and the Healthy People 2020 goal on reducing bullying and identify female students as the priority group.

Olweus concluded that compared with traditional bullying, the prevalence of cyberbullying was actually quite low using the time series data from two large-scale studies in the U.S. and Norway [7]. The average across time prevalences of being bullied verbally and electronically were 17.6 and 4.5% in the U.S. sample (total *n* = 447,000), and 11.0 and 3.4% in Norwegian data (total *n* = 45,000) [7]. However, according to YRBS data, there were 19.70 and 15.44% of high school students who were exposed to traditional bullying and cyberbullying respectively during 2011–2019, indicating that cyberbullying, in the past decade, is not a low frequent phenomenon [20], at least among U.S. adolescents.

In spite of increasing accessibility to smartphones and other internet devices, Olweus found that there was no growth trend in the prevalence of cybervictimization during 2006–2010, neither did the traditional victimization [7, 8]. For cybervictimization, the prevalence ranged from 15.4 to 18.4% in the U.S. and from 10.3 to 11.75% in Norway; for traditional victimization, the results were 15.4–18.4% in the U.S. and 10.3–11.7% in Norway [7]. We achieved a similar conclusion from YRBS data. Among U.S. high school students, the prevalences of cybervictimization and traditional victimization were both unchanged significantly from 2011 to 2019. In the past decade, the prevalence ranged from 14.77 to 16.23% for cybervictimization and from 19.04 to 20.19% for traditional victimization.

Large studies have indicated that there is a substantial overlap between cybervictimization and traditional victimization [21, 22], and the degree of overlap varies from over 90 [7] to 50% [23]. Olweus argued that cyberbullying created a few additional bullying victims, given that the degree of overlap was up to 88% in the U.S. and 93% in Norway [7, 22]. However, YRBS data showed that the degree of overlap was only about 60%, and the overall prevalence of being cyberbullied only was 5.74% during 2011–2019, which meant that contrary to findings from Olweus, cyberbullying actually added a few new victims at least among U.S. adolescents.

Healthy People 2020 provides science-based national objectives for improving the health of Americans during 2011–2020. Healthy People 2020 objective IVP-35 is to reduce the prevalence of traditional victimization in the previous 12 months before the survey among U.S. high school students from 19.9 to 17.9% [13]. However, YRBS data showed that during 2011–2019, the prevalence of traditional victimization ranged from 21.99 to 19.04% and no linear decrease occurred, which suggested that more work is needed to address the issue of school bullying in the next few years. To achieve the Healthy People 2020 bullying goal [24], priority groups should be identified first. Consistent with the results from previous studies in U.S. high school students [14, 18, 25, 26], in our study, female students reported higher prevalences of both cybervictimization and traditional victimization than male peers across the survey cycles, indicating that there are sex disparities in traditional and cyberbullying. The underlying cause for the unchanged trends for both female and male students may be that existing anti-bullying initiatives could reduce verbal and physical bullying effectively, but not relational bullying [19]. Relational bullying victimization ranked as the top bullying form among U.S. adolescents, and female students were more likely to be involved in relational bullying according to the 2010 HBSC U.S. study [27].

Our findings have significant implications for policy and research practice. Policymakers should acknowledge the sex disparities in bullying victimization and give high priority to evidence-based interventions that focus on female students and relational bullying. Given that the prevalence of cybervictimization has remained high and stable among U.S. adolescents during the last decade, researchers need to follow the evolution of bullying in the digital era, investigate its effects on adolescent health, and evaluate the effectiveness of the interventions.

The findings in this study are subject to at least four limitations. First, YRBS data are self-reported, and the experience of being bullied traditionally or electronically may be affected by retrospective recall and social desirability biases [28]. Second, it should be noted that identical or similar measurement properties, including reference period, cutoff point, and context of bullying, must be used to compare the results from different studies. The recommended cutoff for the classification of being bullied is 2 or 3 times per month or more [22]. YRBS used at least once in the past 12 months for the criterion for classification, which led to higher prevalence estimates compared with Olweus’s studies. Third, we measured traditional victimization and cybervictimization by one single item respectively. The use of the single-item measure might possess non-optimal psychometric properties, however, the single-item measure can capture enough information when estimating and comparing the prevalence of bullying victimization [29]. Last, it was not possible to identify the reasons behind the non-declines in school bullying using YRBS data.

## Conclusion

Given that prevalence estimates of bullying victimization unchanged between 2011 and 2019, more work in policies, programs, and practices might be needed in the next few years to reach Healthy People 2020 targets for reducing the bullying disparities among U.S. high school students [24]. And cyberbullying is not a relatively low frequent phenomenon compared with traditional bullying among U.S. high school students, and should not be ignored, especially among female students [30].

## Data Availability

Data is available at https://www.cdc.gov/healthyyouth/data/yrbs/index.htm.

## Abbreviations

HBSC: Health Behaviour in School-aged Children
YRBS: Youth Risk Behavior Survey
OR: Odds ratio
CI: Confidence interval
